# Supplementary figures and images for: Chaperone-Mediated Stress Sensing in Mycobacterium tuberculosis Enables Fast Activation and Sustained Response
Source: mSystems. 2021 Feb 16;6(1):e00979-20. doi: 10.1128/mSystems.00979-20 (PMC8561658; doi:10.1128/mSystems.00979-20)

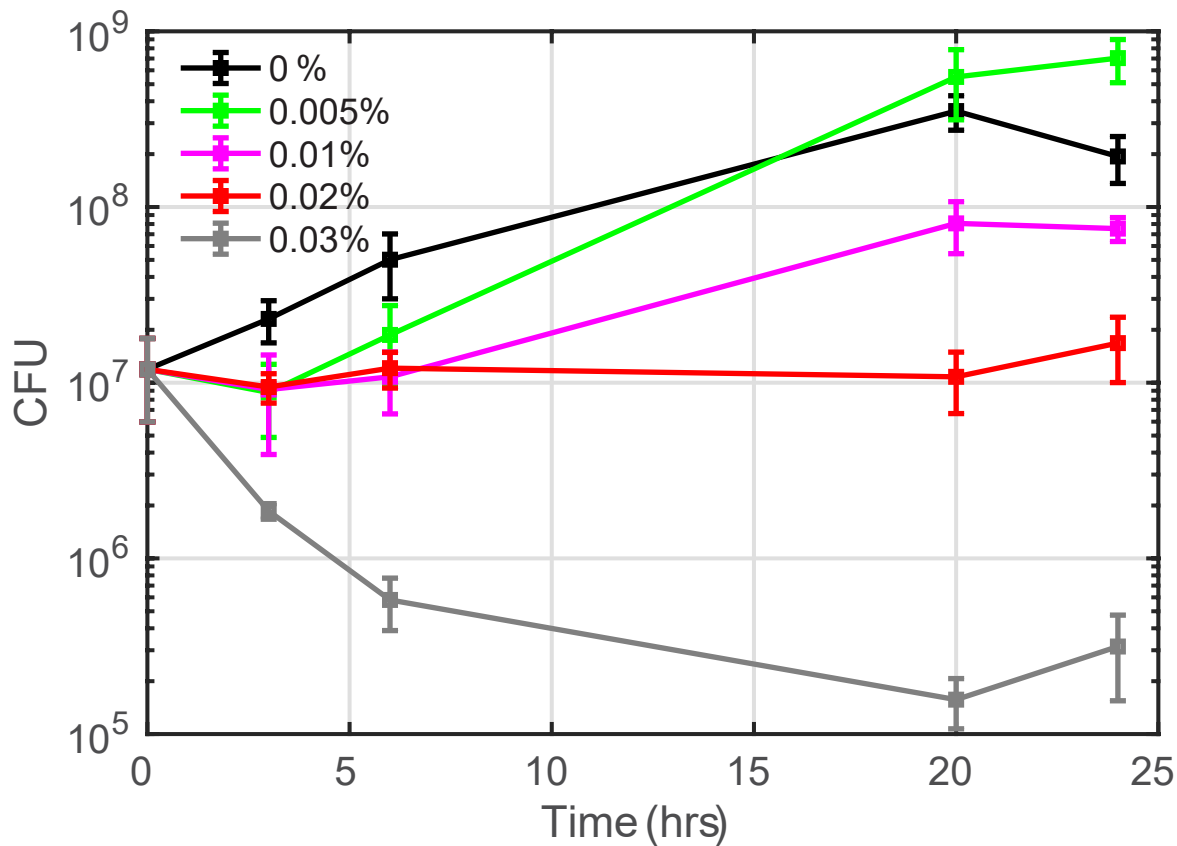

Supplement: FIG S1 [file msystems.00979-20-sf001.pdf]

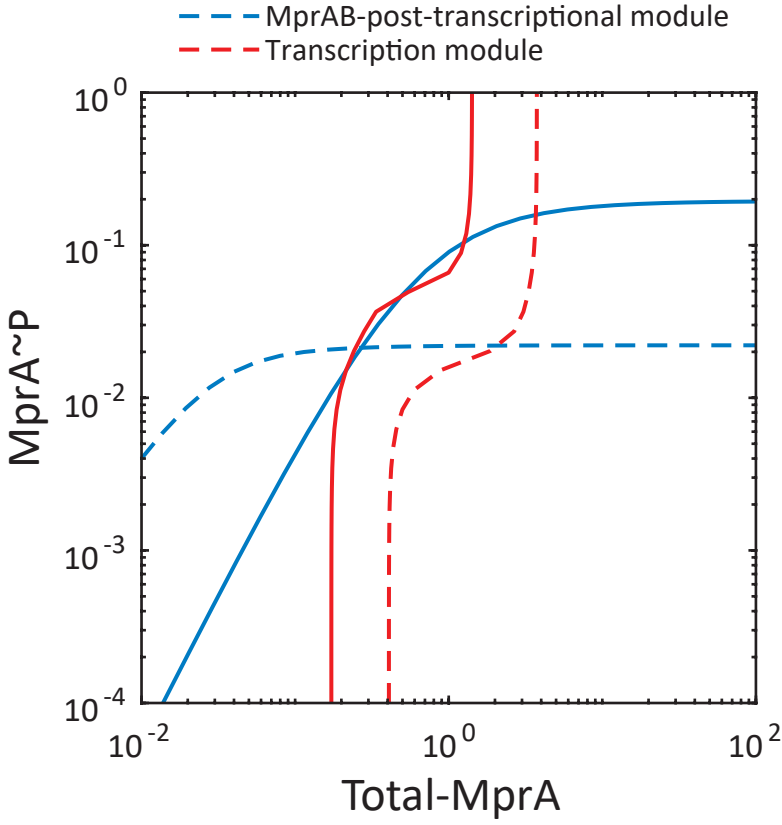

Supplement: FIG S2 [file msystems.00979-20-sf002.pdf]

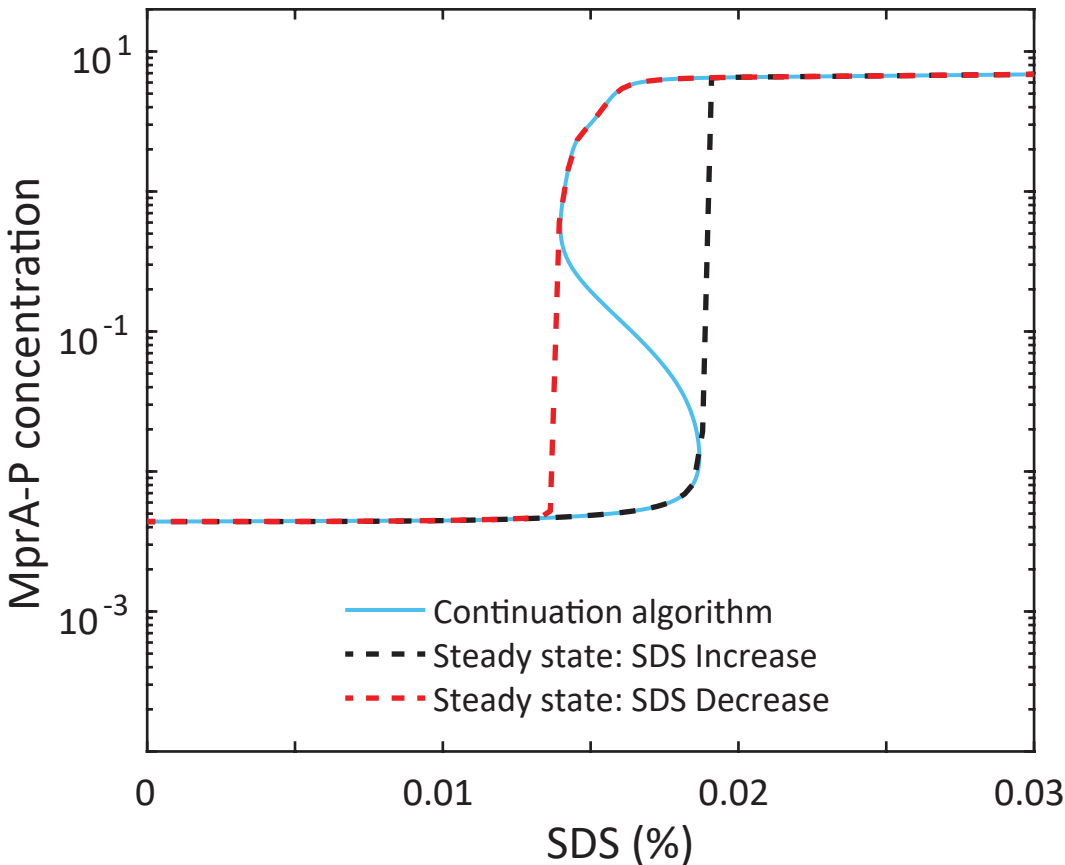

Supplement: FIG S3 [file msystems.00979-20-sf003.pdf]

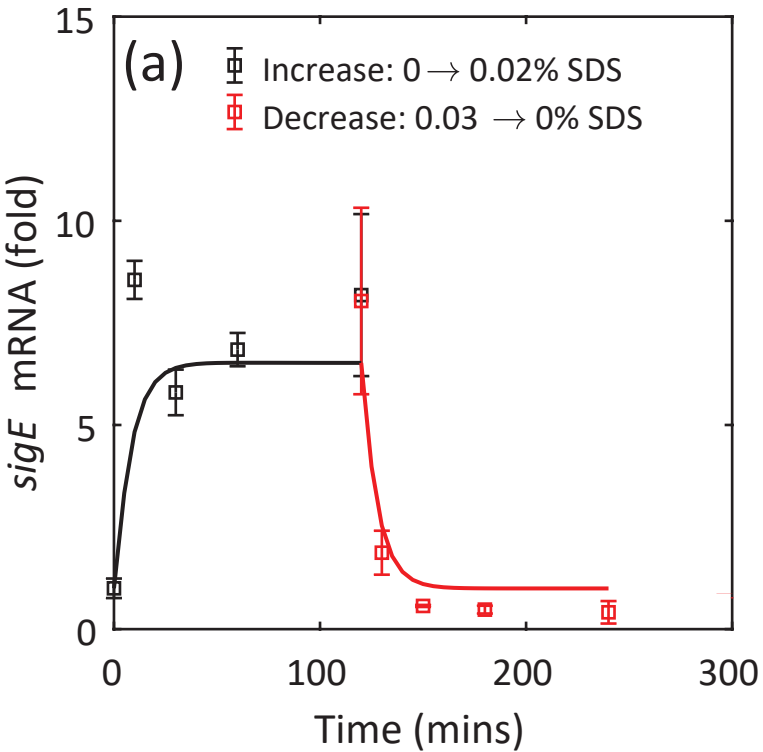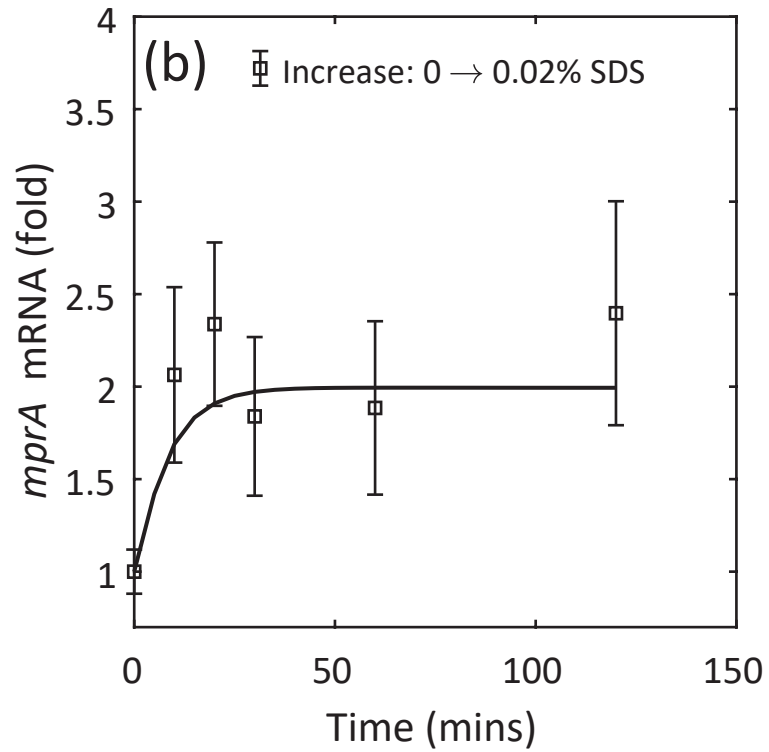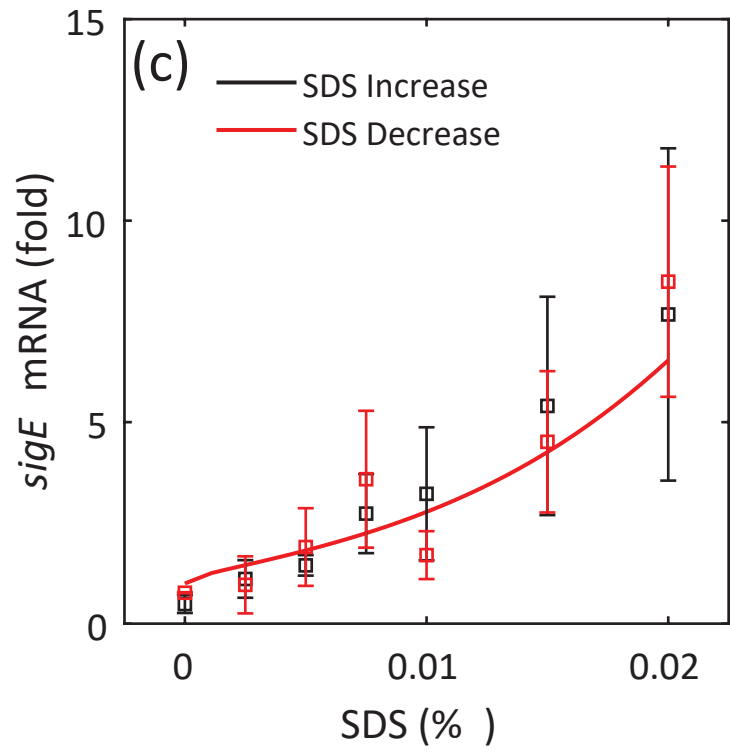

Supplement: FIG S4 [file msystems.00979-20-sf004.pdf]

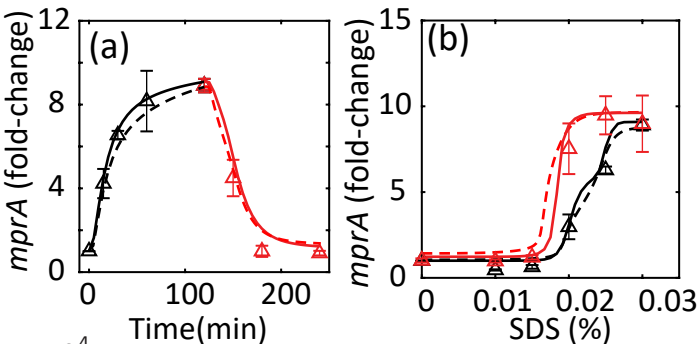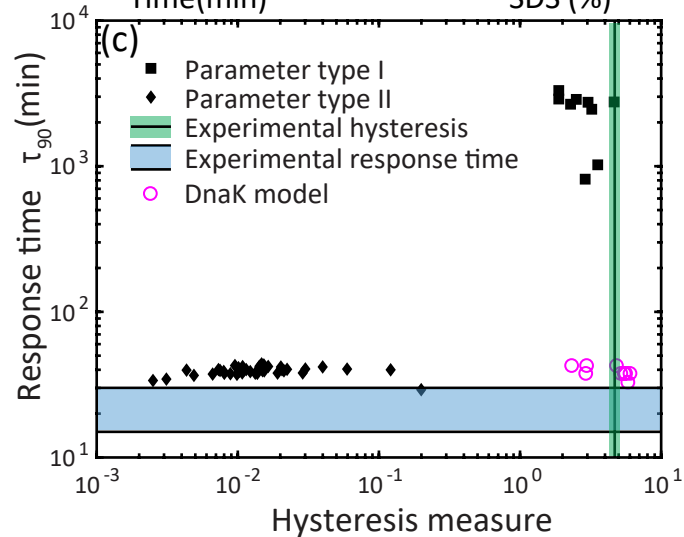

Supplement: FIG S5 [file msystems.00979-20-sf005.pdf]

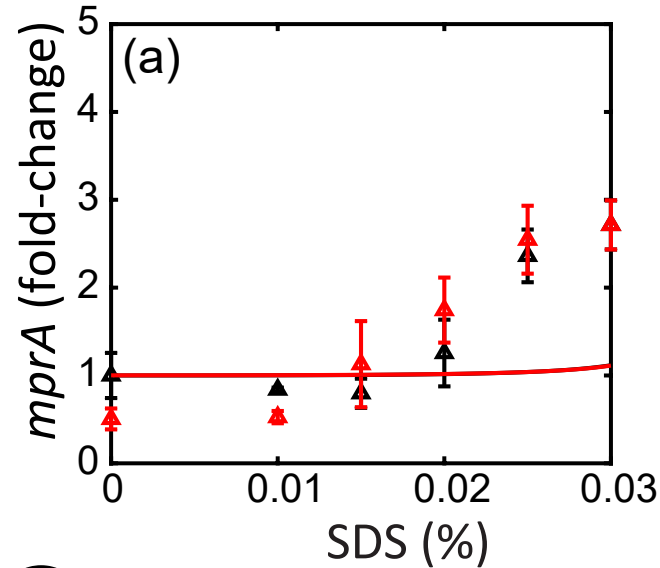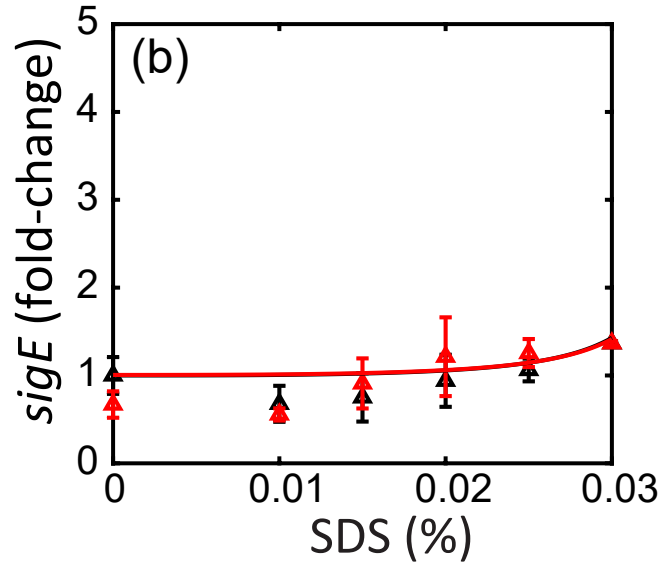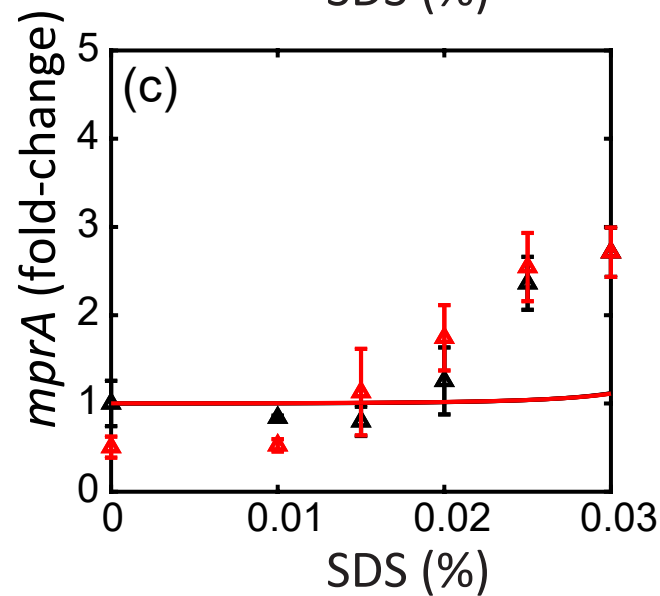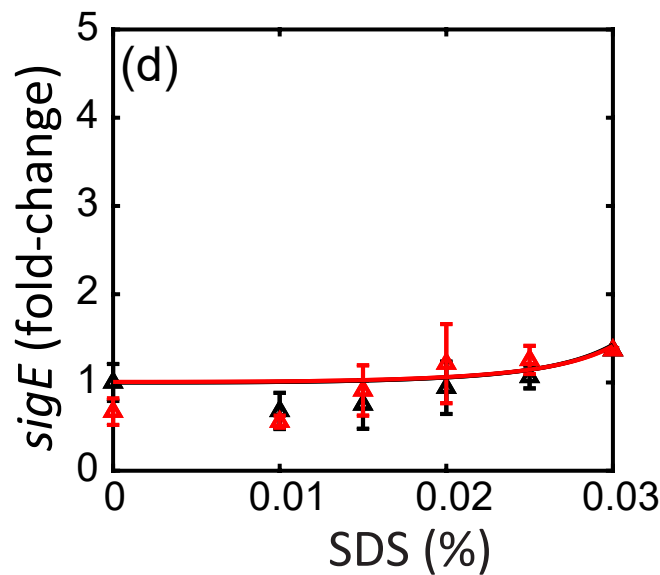

Supplement: FIG S6 [file msystems.00979-20-sf006.pdf]

4 hrs

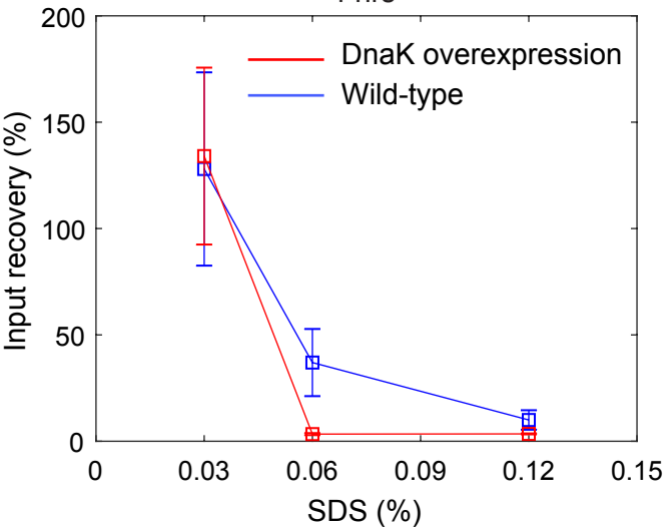

Supplement: FIG S7 [file msystems.00979-20-sf007.pdf]
