# Supplementary material for: Chaperone-Mediated Stress Sensing in Mycobacterium tuberculosis Enables Fast Activation and Sustained Response
Source: mSystems. 2021 Feb 16;6(1):e00979-20. doi: 10.1128/mSystems.00979-20 (PMC8561658; doi:10.1128/mSystems.00979-20)
Supplement: TABLE S1 [file msystems.00979-20-st001.pdf]

TableS1: Primers for construction of P_{mprA}-*dnaK* fusion

Gene	Forward (5'-3')	Reverse (5'-3')
P_{mprA}	ATAATCTAGACCGCCGCCGCGGTGTTGG	ATATTCTAGACACGGACACCAGTGTCGTC
dnaK	ATATTCTAGAGCTCGTGCGGTCGGGAT	ATATTAAGCTTCTCGGGATCGATCCGCC

*Restriction recognition sites are underlined
