# Supplementary material for: Chaperone-Mediated Stress Sensing in Mycobacterium tuberculosis Enables Fast Activation and Sustained Response
Source: mSystems. 2021 Feb 16;6(1):e00979-20. doi: 10.1128/mSystems.00979-20 (PMC8561658; doi:10.1128/mSystems.00979-20)
Supplement: TEXT S1 [file msystems.00979-20-s0001.pdf]

Supporting Material for: Chaperone-mediated stress-sensing in *Mycobacterium tuberculosis* enables fast activation and sustained response

Satyajit D. Rao[1,a], Pratik Datta[2,a], Maria Laura Gennaro[2], Oleg A. Igoshin[1*]

Model reactions

Transcription
Transcription of *mprAB* and *sigE* is modeled using average promoter occupancy at equilibrium. Transcription occurs at two different rates depending on the binding state of the promoter – basal rate if the promoter is empty, and activated rate if the promoter is bound by a transcription factor. Thus the net rate of production of mRNA $= k_{basal}(1-\rho) + k_{act}\rho$, where ρ is the occupancy fraction of the promoter. All mRNA is assumed to be actively degraded with a rate k_{mdeg}.

The *mprAB* operon is transcribed from two promoters: P1 – MprA-P activated promoter, P2 – σ^Eactivated promoter.

- Mean P1 occupancy fraction (by MprA-P) $= \frac{\left(\frac{\text{MprA-P}}{K_1}\right)^2}{1+\left(\frac{\text{MprA-P}}{K_1}\right)^2+\left(\frac{\text{MprA}}{K_{1b}}\right)^2}$
- Mean P1 occupancy fraction (by MprA) $= \frac{\left(\frac{\text{MprA}}{K_{1b}}\right)^2}{1+\left(\frac{\text{MprA-P}}{K_1}\right)^2+\left(\frac{\text{MprA}}{K_{1b}}\right)^2}$
- Mean P2 occupancy fraction $= \frac{\left(\frac{\sigma^E}{K_2}\right)}{1+\left(\frac{\sigma^E}{K_2}\right)}$

The *sigE* gene is transcribed from one MprA-P dependent promoter (P_E)

- Mean P_E occupancy fraction (by MprA-P) $= \frac{\left(\frac{\text{MprA-P}}{K_3}\right)^2}{1+\left(\frac{\text{MprA-P}}{K_3}\right)^2+\left(\frac{\text{MprA}}{K_{3b}}\right)^2}$

- Mean P_E occupancy fraction (by MprA) $= \frac{\left(\frac{\text{MprA}}{K_{3b}}\right)^2}{1+\left(\frac{\text{MprA-P}}{K_3}\right)^2+\left(\frac{\text{MprA}}{K_{3b}}\right)^2}$

Translation

MprA,MprB: Both proteins are translated from the same message at different rates

$mprAB \xrightarrow{\text{k}_{\text{tlnA}}} mprAB + \text{MprA}$

$mprAB \xrightarrow{\text{k}_{\text{tlnB}}} mprAB + \text{MprB}$

σ^E: $sigE \xrightarrow{\text{k}_{\text{tlnE}}} sigE + \sigma^E$

RseA: Transcription and translation is bundled into a single step

$\phi \xrightarrow{\text{k}_{\text{tlnR}}} \text{RseA}$

DnaK: Transcription and translation is bundled into a single step

$\phi \xrightarrow{\text{k}_{\text{tlnD}}} \text{DnaK}$

DnaK in overexpressing mutant: Additional DnaK is synthesized from $mdnaK = mprAB$

$mdnaK \xrightarrow{\beta} mdnaK + \text{DnaK}$

Post-translational interactions

Signal sensing

1. In the simple model of MprB activation, MprB switches from phosphatase dominant (MprB) form, as it is in by default under unstressed condition, to kinase dominant (MprB*) form.

   $\text{MprB} \underset{\text{k}_{-1}}{\overset{\text{k}_1}{\rightleftharpoons}} \text{MprB}^*$

2. In DnaK-dependent activation model, instead of a first order activation of MprB, the DnaK-model includes a second order reaction, where MprB (kinase) binds DnaK into a complex (phosphatase)

   $\mathrm{MprB + DnaK \underset{k_{bdb}}{\overset{k_{bdf}}{\rightleftharpoons}} [MprB \cdot DnaK]}$

   In presence of SDS, unfolded protein load (UF) builds up, leading to DnaK switching away from binding MprB to binding unfolded proteins:

   $\mathrm{UF + DnaK \underset{k_{dsb}}{\overset{k_{dsf}}{\rightleftharpoons}} [UF \cdot DnaK]}$

3. RseA degradation rate k_{rd} increases as a function of signal

Equations for signal sensing parameters as a function of SDS:

$$
\begin{aligned}
k_1 &= k_1(0) + k_{1,max}\left(\frac{\mathrm{SDS}^{n1}}{K_1^{n1} + \mathrm{SDS}^{n1}}\right) \\
k_{rd} &= k_{rd}(0) + k_{rd,max}\left(\frac{\mathrm{SDS}^{n2}}{K_2^{n2} + \mathrm{SDS}^{n2}}\right) \\
UF &= UF_{max}\left(\frac{\mathrm{SDS}^{n3}}{K_3^{n3} + \mathrm{SDS}^{n3}}\right)
\end{aligned}
\tag{1}
$$

For simple MprAB model:
$k_1(0) = 4.3 \times 10^{-4}$, $k_{1,max} = 0.054$, $K_1 = 0.01$, $n1 = 3$
$k_{rd}(0) = 6.3 \times 10^{-5}$, $k_{rd,max} = 0.02$, $K_2 = 0.048$, $n2 = 6.5$

For DnaK-MprAB model:
$k_{rd}(0) = 9.6 \times 10^{-5}$, $k_{rd,max} = 9.6 \times 10^{-3}$, $K_2 = 0.023$, $n2 = 9.4$
$UF_{max} = 13\mu M$, $K_3 = 0.08$, $n3 = 2.8$

MprA-MprB reactions

1. MprB* autophosphorylation.

   $\mathrm{MprB^* \underset{k_{-2}}{\overset{k_2}{\rightleftharpoons}} MprB{-}P}$

2. Phosphotransfer to MprA

   $\mathrm{MprB{-}P + MprA \underset{k_{-3}}{\overset{k_3}{\rightleftharpoons}} [MprB{-}P \cdot MprA] \xrightarrow{k_4} MprB^{*+} MprA{-}P}$

3. MprB dephosphorylates MprA-P

$$\mathrm{MprB} + \mathrm{MprA{-}P} \underset{k_{-5}}{\overset{k_5}{\rightleftharpoons}} [\mathrm{MprB} \cdot \mathrm{MprA{-}P}] \xrightarrow{k_6} \mathrm{MprB} + \mathrm{MprA}$$

σ^E-RseA reactions

1. Anti-sigma factor RseA regulates σ^Eactivity by forming a complex

$$\sigma^{\mathrm{E}} + \mathrm{RseA} \underset{k_d}{\overset{k_b}{\rightleftharpoons}} [\sigma^{\mathrm{E}} \cdot \mathrm{RseA}]$$

Model ODEs

Model with simple activation mechanism of MprAB

$$\frac{d[A]}{dt} = k_{tlnA} mAB - k_3 B_P A + k_{-3}[B_P.A] + k_6[B.A_P] - k_{pdeg} A$$

$$\frac{d[A_P]}{dt} = k_4[B_P.A] - k_5 B A_P + k_{-5}[B.A_P] - k_{pdeg} A_P$$

$$\frac{d[B^*]}{dt} = k_1 B - k_{-1} B^* - k_2 B^* + k_{-2} B_P + k_4[B_P.A] - k_{pdeg} B^*$$

$$\frac{d[B]}{dt} = k_{tlnB} mAB + k_{-1} B^* - k_1 B - k_5 B A_P + k_{-5}[B.A_P] + k_6[B.A_P] - k_{pdeg} B$$

$$\frac{d[B_P]}{dt} = k_2 B^* - k_{-2} B_P + k_{-3}[B_P.A] - k_3 B_P A - k_{pdeg} B_P$$

$$\frac{d[B_P.A]}{dt} = k_3 B_P A - k_{-3}[B_P.A] - k_4[B_P.A] - k_{pdeg}[B_P.A]$$

$$\frac{d[B.A_P]}{dt} = k_5 B A_P - k_{-5}[B.A_P] - k_6[B.A_P] - k_{pdeg}[B.A_P]$$

$$\frac{d[E]}{dt} = k_{tlnE} mE - k_b E R + k_d[ER] + k_{rd}[ER] - k_{pdeg}[E]$$

$$\frac{d[R]}{dt} = k_{tlnR} - k_b E R + k_d[ER] - k_{rd}[R] - k_{pdeg}[R]$$

$$\frac{d[ER]}{dt} = k_b E R - k_d[ER] - k_{rd}[ER] - k_{pdeg}[ER]$$

$$\frac{d[mAB]}{dt} = k_{btpn1} \frac{1 + f_1\left(\frac{A_P}{K_1}\right)^2 + f_{1b}\left(\frac{A}{K_{1b}}\right)^2}{1 + \left(\frac{A_P}{K_1}\right)^2 + \left(\frac{A}{K_{1b}}\right)^2} - k_{mdeg} mAB$$

$$\frac{d[mE]}{dt} = k_{btpn3} \frac{1 + f_3\left(\frac{A_P}{K_3}\right)^2 + f_{3b}\left(\frac{A}{K_{3b}}\right)^2}{1 + \left(\frac{A_P}{K_3}\right)^2 + \left(\frac{A}{K_{3b}}\right)^2} + k_{btpn2} \frac{f_2\left(\frac{E}{K_2}\right)}{1 + \left(\frac{E}{K_2}\right)} - k_{mdeg} mE$$

Model with DnaK-dependent activation of MprAB

$$\frac{d[A]}{dt} = k_{tlnA}mAB - k_3B_PA + k_{-3}[B_P.A] + k_6[BD.A_P] - k_{pdeg}A$$
$$\frac{d[A_P]}{dt} = k_4[B_P.A] - k_5BDA_P + k_{-5}[BD.A_P] - k_{pdeg}A_P$$
$$\frac{d[UF]}{dt} = +k_{pdeg}[D.UF] - k_{dsf}DUF + k_{dsb}[D.UF]$$
$$\frac{d[D.UF]}{dt} = k_{dsf}DUF - k_{dsb}[D.UF] - k_{pdeg}[D.UF]$$
$$\frac{d[D]}{dt} = k_{tlnD} - k_{bdf}BD + k_{bdb}BD - k_{dsf}DUF + k_{dsb}[D.UF] - k_{pdeg}D$$
$$\frac{d[BD]}{dt} = k_{bdf}BD - k_{bdb}[BD] - k_{pdeg}B^* - k_5BDA_P + k_{-5}[BD.A_P] + k_6[BD.A_P] - k_{pdeg}[BD]$$
$$\frac{d[B]}{dt} = k_{tlnB}mAB - k_2B^* + k_{-2}B_P + k_4[B_P.A] - k_{bdf}BD + k_{bdb}[BD] - k_{pdeg}B$$
$$\frac{d[B_P]}{dt} = k_2B^* - k_{-2}B_P + k_{-3}[B_P.A] - k_3B_PA - k_{pdeg}B_P$$
$$\frac{d[B_P.A]}{dt} = k_3B_PA - k_{-3}[B_P.A] - k_4[B_P.A] - k_{pdeg}[B_P.A]$$
$$\frac{d[BD.A_P]}{dt} = k_5BDA_P - k_{-5}[BD.A_P] - k_6[BD.A_P] - k_{pdeg}[BD.A_P]$$
$$\frac{d[E]}{dt} = k_{tlnE}mE - k_bER + k_d[ER] + k_{rd}[ER] - k_{pdeg}[E]$$
$$\frac{d[R]}{dt} = k_{tlnR} - k_bER + k_d[ER] - k_{rd}[R] - k_{pdeg}[R]$$
$$\frac{d[ER]}{dt} = k_bER - k_d[ER] - k_{rd}[ER] - k_{pdeg}[ER]$$
$$\frac{d[mAB]}{dt} = k_{btpn1}\frac{1+f_1\left(\frac{A_P}{K_1}\right)^2+f_{1b}\left(\frac{A}{K_{1b}}\right)^2}{1+\left(\frac{A_P}{K_1}\right)^2+\left(\frac{A}{K_{1b}}\right)^2} - k_{mdeg}mAB$$
$$\frac{d[mE]}{dt} = k_{btpn3}\frac{1+f_3\left(\frac{A_P}{K_3}\right)^2+f_{3b}\left(\frac{A}{K_{3b}}\right)^2}{1+\left(\frac{A_P}{K_3}\right)^2+\left(\frac{A}{K_{3b}}\right)^2} + k_{btpn2}\frac{f_2\left(\frac{E}{K_2}\right)}{1+\left(\frac{E}{K_2}\right)} - k_{mdeg}mE$$

Model Parameters

Model with simple activation mechanism of MprAB

Parameter	Description	Units	Value
k_{btpn1}	Basal transcription rate of *sigE*	μMs^{-1}	1.43×10^{-6}
k_{btpn3}	Basal transcription rate of *mprA* from MprA-promoter	μMs^{-1}	8.1×10^{-7}
k_{btpn2}	Basal transcription rate of *mprA* from σ^E-promoter	μMs^{-1}	1.7×10^{-6}
f_1	Fold increase in *mprA* transcription rate due to MprA-P	-	2.8
f_2	Fold increase in *mprA* transcription rate due to σ^E	-	6.6

f_3	Fold increase in *sigE* transcription rate due to MprA-P	-	10.5
K_1	Dissociation constant for MprA-P binding *mprA* promoter	μM	0.04
K_2	Dissociation constant for σ^Ebinding *mprA* promoter	μM	0.035
K_3	Dissociation constant for MprA-P binding *sigE* promoter	μM	0.04
k_{tlnB}	MprB translation rate	s^{-1}	2.5×10^{-3}
k_{tlnA}	MprA translation rate	s^{-1}	1.1×10^{-2}
k_{tlnE}	σ^E translation rate	s^{-1}	2.2×10^{-2}
k_{tlnR}	RseA synthesis rate	$\mu M s^{-1}$	2.6×10^{-5}
k_{pdeg}	Protein degradation+dilution rate	s^{-1}	7.7×10^{-5}
k_{mdeg}	mRNA degradation rate	s^{-1}	1.2×10^{-3}
$k_1(0)$	MprB activation rate at 0 SDS	s^{-1}	4.3×10^{-4}
k_{-1}	MprB* deactivation rate	s^{-1}	3.9×10^{-2}
k_2	MprB* autophosphorylation rate	s^{-1}	7.7×10^{-3}
k_{-2}	MprB-P auto-dephosphorylation rate	s^{-1}	3.6×10^{-5}
k_3	MprB-P-MprA association rate	$\mu M^{-1} s^{-1}$	0.01
k_{-3}	MprB-P-MprA dissociation rate	s^{-1}	2.25
k_4	MprB-P-MprA phosphotransfer rate	s^{-1}	0.044
k_5	MprB-MprA-P association rate	$\mu M^{-1} s^{-1}$	0.0698
k_{-5}	MprB-MprA-P dissociation rate	s^{-1}	1.8×10^{-3}
k_6	MprB-MprA-P dephosphorylation rate	s^{-1}	4.4×10^{-3}
k_b	σ^E-RseA association rate	$\mu M^{-1} s^{-1}$	9.7×10^0
k_d	σ^E-RseA dissociation rate	s^{-1}	8.5×10^{-4}
k_{rd}	RseA proteolysis rate at 0 SDS	s^{-1}	6.3×10^{-5}

Model with DnaK-dependent activation of MprAB

Parameter	Description	Units	Value
k_{btpn1}	Basal transcription rate of *sigE*	$\mu M s^{-1}$	1.0×10^{-6}

k_{btpn3}	Basal transcription rate of *mprA* from MprA-promoter	$\mu M s^{-1}$	6.5×10^{-7}
k_{btpn2}	Basal transcription rate of *mprA* from σ^E-promoter	$\mu M s^{-1}$	1.2×10^{-6}
f_1	Fold increase in *mprA* transcription rate due to MprA-P	-	5
f_2	Fold increase in *mprA* transcription rate due to σ^E	-	7.5
f_3	Fold increase in *sigE* transcription rate due to MprA-P	-	20
K_1	Dissociation constant for MprA-P binding *mprA* promoter	μM	0.026
K_2	Dissociation constant for σ^Ebinding *mprA* promoter	μM	0.23
K_3	Dissociation constant for MprA-P binding *sigE* promoter	μM	0.026
k_{tlnB}	MprB translation rate	s^{-1}	6.0×10^{-3}
k_{tlnA}	MprA translation rate	s^{-1}	2.0×10^{-2}
k_{tlnE}	σ^E translation rate	s^{-1}	5.4×10^{-3}
k_{tlnR}	RseA synthesis rate	$\mu M s^{-1}$	2.7×10^{-4}
k_{tlnD}	DnaK synthesis rate	$\mu M s^{-1}$	3.05×10^{-5}
k_{pdeg}	Protein degradation+dilution rate	s^{-1}	3.8×10^{-5}
k_{mdeg}	mRNA degradation rate	s^{-1}	1.2×10^{-3}
k_{bdf}	MprB-DnaK association rate	$\mu M^{-1} s^{-1}$	0.55
k_{bdb}	MprB-DnaK dissociation rate	s^{-1}	7.7×10^{-4}
k_{dsf}	MprB-UF association rate	$\mu M^{-1} s^{-1}$	0.1
k_{dsb}	MprB-UF dissociation rate	s^{-1}	4.1×10^{-4}
k_2	MprB* autophosphorylation rate	s^{-1}	0.08
k_{-2}	MprB-P auto-dephosphorylation rate	s^{-1}	5.7×10^{-5}
k_3	MprB-P-MprA association rate	$\mu M^{-1} s^{-1}$	0.77
k_{-3}	MprB-P-MprA dissociation rate	s^{-1}	0.0394
k_4	MprB-P-MprA phosphotransfer rate	s^{-1}	0.02
k_5	MprB-MprA-P association rate	$\mu M^{-1} s^{-1}$	0.036

k_{-5}	MprB-MprA-P dissociation rate	s^{-1}	4.16×10^{-4}
k_6	MprB-MprA-P dephosphorylation rate	s^{-1}	0.04
k_b	σ^E-RseA association rate	$\mu M^{-1} s^{-1}$	2.88
k_d	σ^E-RseA dissociation rate	s^{-1}	7.5×10^{-3}
k_{rd}	RseA proteolysis rate at 0 SDS	s^{-1}	9.6×10^{-5}
